# The Clinical Usefulness of Evaluating the Lens and Intraocular Lenses Using Optical Coherence Tomography: An Updated Literature Review

**DOI:** 10.3390/jcm13237070

**Published:** 2024-11-22

**Authors:** José Ignacio Fernández-Vigo, Lucía De-Pablo-Gómez-de-Liaño, Ignacio Almorín-Fernández-Vigo, Beatriz De-Pablo-Gómez-de-Liaño, Ana Macarro-Merino, Julián García-Feijóo, José Ángel Fernández-Vigo

**Affiliations:** 1 Ophthalmology Department, Hospital Clínico San Carlos, Instituto de Investigación Sanitaria (IdISSC), 28040 Madrid, Spain; 2 Department of Immunology, Ophthalmology and ENT, Faculty of Medicine, Universidad Complutense de Madrid, 28040 Madrid, Spain; 3 Centro Internacional de Oftalmología Avanzada, 28010 Madrid, Spain; 4 Ophthalmology Department, Hospital Universitario 12 de Octubre, 28041 Madrid, Spain; 5 Department of Immunology, Ophthalmology and ENT, Faculty of Optics, Universidad Complutense de Madrid, 28040 Madrid, Spain; 6 Centro Internacional de Oftalmología Avanzada, 06011 Badajoz, Spain; 7 Ophthalmology Department, Hospital Universitario Central de la Cruz Roja San José y Santa Adela, 28003 Madrid, Spain; 8 Department of Ophthalmology, Faculty of Medicine, University of Extremadura, 06006 Badajoz, Spain

**Keywords:** optical coherence tomography, intraocular lens, lens, cataract, intraocular lens opacification

## Abstract

The Lens Dysfunction Syndrome includes two widespread ocular disorders: presbyopia and cataract. Understanding its etiology, onset, progression, impact, prevention, and treatment remains a significant scientific challenge. The lens is a fundamental structure of the ocular dioptric system that allows for focus adjustment or accommodation to view objects at different distances. Its opacification, primarily related to aging, leads to the development of cataracts. Traditionally, lens alterations have been diagnosed using a slit lamp and later with devices based on the Scheimpflug camera. However, both methods have significant limitations. In recent years, optical coherence tomography (OCT) has become a valuable tool for assessing the lens and pseudophakic intraocular lenses (IOLs) in clinical practice, providing a highly detailed non-invasive evaluation of these structures. Its clinical utility has been described in assessing the shape, location or position, and size of the lens, as well as in determining the degree and type of cataract and its various components. Regarding pseudophakic IOLs, OCT allows for the accurate assessment of their position and centering, as well as for detecting possible complications, including the presence of glistening or IOL opacification. Furthermore, OCT enables the evaluation of the posterior capsule and its associated pathologies, including late capsular distension syndrome. This review highlights the key applications of OCT in the assessment of the lens and pseudophakic IOLs.

## 1. Introduction

The lens is a transparent, biconvex structure positioned behind the iris and anterior to the vitreous body, functioning as a key element of the ocular dioptric system. Its principal role is to refract and focus light onto the retina to form a clear image. Additionally, it can adjust its focus, or accommodate, to enable vision of objects at varying distances [1]. With aging, the lens may become opaque, leading to the development of cataracts, which impair both the quality and quantity of vision in patients and are among the primary causes of blindness worldwide. The standardized approach for cataract treatment involves their surgical removal through phacoemulsification followed by the implantation of a pseudophakic intraocular lens (IOL), ideally implanted within the lens capsular bag [1,2].

Traditionally, the diagnosis and monitoring of lens and IOL abnormalities has been performed by slit-lamp biomicroscopy [2]. However, despite the introduction of diverse grading scales, this technique has difficulties in establishing reproducible classifications of lens and IOL pathologies due mainly to its subjectivity. Furthermore, this issue also complicates the assessment of changes over time [2].

The advancement of technology has favored the development of devices designed to assess intraocular structures (i.e., biometrics). These initial devices used an infrared diode laser with a wavelength ranging from 750 to 820 nm [3], which is projected through the ocular media and subsequently reflected off the internal limiting membrane of the retina. The reflected signal is collected and analyzed to provide accurate measurements of axial length, lens thickness, and other key ocular parameters. This technology allows biometric calculations with exceptional reproducibility, which is crucial for the preoperative planning of cataract surgeries and for determining IOL power. However, this technique has certain limitations in the study of the lens, such as its inability to provide depth imaging of the lens and surrounding structures, a lack of detail and resolution in evaluating the internal layers of the lens, and difficulty in detecting subtle pathologies or changes in lens density [2].

Subsequently, other imaging techniques were developed, including devices based on the Scheimpflug camera and ultrasound biomicroscopy, which have been used to assess the lens and IOLs both qualitatively and quantitatively. However, these techniques have certain limitations regarding resolution and penetration, hindering a comprehensive and detailed assessment of the internal structure of the lens and IOLs, as well as their interaction with surrounding structures [2,4,5]. Additionally, while ultrasound biomicroscopy (UBM) offers high image penetration, it has several drawbacks, including the necessity for an experienced examiner, patient discomfort, and the requirement for direct contact.

In this context, optical coherence tomography (OCT) has emerged in recent years as a promising alternative for analyzing the anterior segment of the eye [6], allowing a more detailed and non-invasive assessment of the lens and IOLs. Consequently, OCT has facilitated significant advancements in the understanding, diagnosis, treatment, and complications of lens and IOL pathologies.

Through an extensive literature review and case presentations, this article aimed to analyze how OCT has revolutionized the field of ophthalmology, particularly concerning the lens and IOLs. This review compiles existing knowledge and underscores the practical applications of OCT in routine clinical practice.

## 2. Methods

A comprehensive but non-systematic review of the literature in PubMed (www.pubmed.gov, accessed on 1 August 2024) was performed. We carried out literature research using the following keywords: “Optical coherence tomography” OR “OCT” AND “intraocular lens” OR “crystalline lens” OR “Lens” OR “Cataract”. Studies and publications considered as relevant by the authors’ discretion, of a high quality and with robust scientific evidence, were included in the review. Additionally, we conducted a manual review of the reference lists from the different studies included in this review to identify any supplementary publications that may have contributed valuable insights to the current paper.

## 3. Results

### 3.1. Optical Coherence Tomography (OCT)

**Figure 1:**
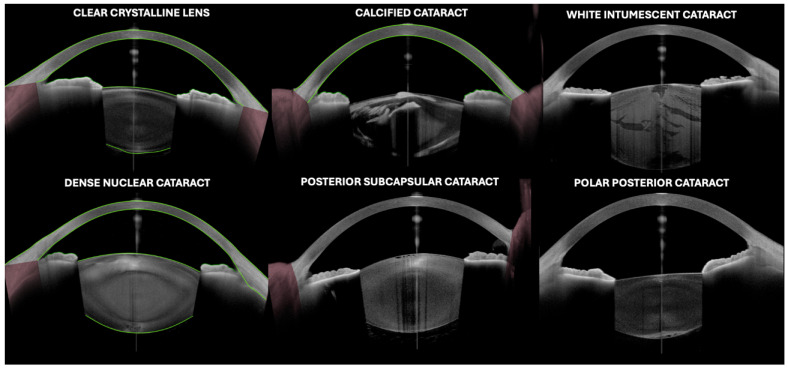
An image of the lens by optical coherence tomography (Anterion AS-OCT, Heidelberg engineering, Heidelberg, Germany) in different clinical scenarios.

OCT has been established as a non-invasive, high-resolution imaging tool in the field of ophthalmology. It uses low-coherence interferometry to generate cross-sectional images of tissues, enabling detailed visualization of ocular structures in vivo [6]. Initially used for the study of the retina and optic nerve, its application has significantly expanded to include analysis of the anterior segment of the eye [6], both of the lens and IOLs (Figure 1). OCT technology has evolved to swept-source OCT (SS-OCT), which uses wavelengths around 1310 nm for the anterior segment, allowing deeper penetration into denser tissues such as the lens and providing detailed imaging of the iridocorneal angle and IOLs. Consequently, it has become an essential tool for research and clinical monitoring in cataract surgery and IOL management.

Different OCT devices are currently market available that allow the assessment of both lens and pseudophakic IOLs. However, visualization depends on the technical specifications of the device and the different scanning protocols each device allows.

### 3.2. Clinical Usefulness of OCT in Lens Examination

**Figure 2:**
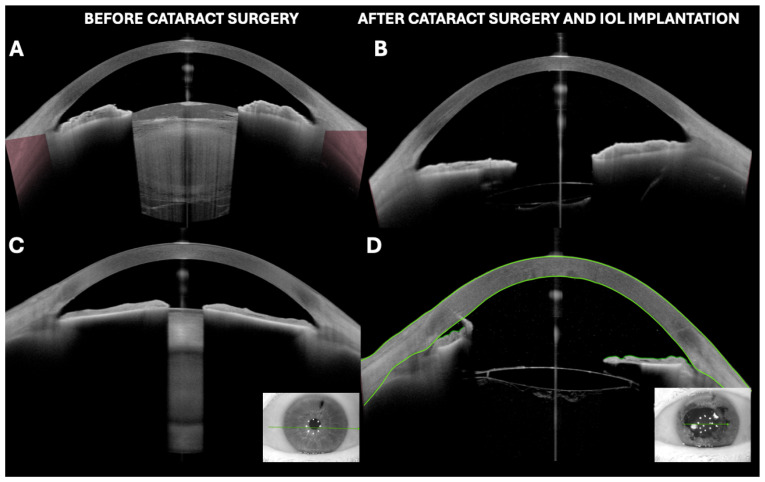
(**A**,**B**) White intumescent cataract visualized by OCT before and after phacoemulsification surgery with intraocular lens implantation. (**C**,**D**) Dense nuclear cataract with irido-lenticular synechiae in miosis and its appearance after cataract surgery with phacoemulsification, intraocular lens implantation, and iridial stretching maneuvers. Irido-endothelial synechia at main incision site can be observed. All images were taken with Anterion AS-OCT (Heidelberg engineering, Heidelberg, Germany).

Over the last several years, different applications of OCT for lens evaluation have been reported (Table 1, Figure 1 and Figure 2).

### 3.3. Evaluation of Lens Morphology (Lens Biometry)

#### 3.3.1. Lens Biometry

Recent technological advancements in anterior segment visualization and measurement have led to the optimization of intraocular lens power calculation. Currently, OCT-based biometers provide an improved measurement rate of intraocular structures and distances, even in the presence of media opacities, reducing the measurement error rate from 11.12% in previous infrared coherent light-based devices to the current 1.51% with OCT [7]. This has also enabled the use of more predictive variables to better adjust to the patient’s biometric profile. In this regard, lens thickness (LT) measurement has gained significant importance in recent years [8]. Anterior segment OCT (AS-OCT) has allowed LT measurements that are statistically comparable to A-scan ultrasonography, without the need for contact, and in a faster and more efficient manner [9].

#### 3.3.2. Lens Positioning (i.e., Decentration, Tilt, Dislocations, Etc.)

The proper centering of IOLs, particularly multifocal ones, is crucial to ensuring optimal postoperative visual function. A significant decentration of a multifocal IOL relative to the visual axis can result in central light passing through the diffractive rings but not the center of the optical zone, potentially leading to high-order aberrations that can significantly impact patient satisfaction [10,11].

The angle kappa is defined as the potential difference between the pupillary center and the visual axis, while the angle alpha is defined as the potential difference between the optical axis and the visual axis. Avoiding those angles being large is preferred for the preoperative selection of candidates for multifocal IOLs in clinical practice, as it has been postulated that larger angles could impair visual results. However, the calculation of the angle alpha assumes that the central point of the limbal plane coincides with the central point of the IOL plane. In this regard, Wang et al., using SS-OCT, observed a natural tilt of the lens relative to the visual axis of approximately 3.7° ± 1.1°. Therefore, the magnitude of the tilt and decentration of the lens relative to the visual axis may be significant [10]. Dong et al. have also investigated lens tilt and decentration relative to the corneal vertex (CV) using SS-OCT. They found that the mean distance between the center points of the lens plane and the limbal plane was approximately 0.33 ± 0.18 mm. The distance of the center of the limbal plane from the CV was approximately 0.31 ± 0.14 mm, which was similar to the distance of the center of the lens plane from the CV (0.33 ± 0.20 mm; *p* = 0.354). However, in 80% of eyes (24/30), the centers of the limbal and lens planes were in different quadrants. In addition, the lens was tilted approximately 4.16° ± 1.97° relative to the CV. Therefore, the authors concluded that the center of the limbal plane did not align with that of the lens plane. Lens tilt and decentration were naturally observed phenomena relative to the CV.

There is much debate, and several authors have suggested that the definition of the angle kappa used for screening premium IOLs needs to be updated [11,12]. Ideally, the angle alpha should represent the difference between the center of the IOL and the visual axis.

The presence of the iris complicates the complete visualization of the lens with most available imaging devices. Therefore, some devices use the center of the distance to the limbus as the lens center to calculate the angle alpha. In this regard, the Anterion SS-OCT, along with the 3D reconstruction method, has demonstrated its capability to facilitate an accurate calculation of the lens center and verify the potential difference between the limbus center and the lens center. In this study, the lens center and the limbus center were found to be different and separated by a distance of 0.33 ± 0.19 mm. Furthermore, although the distance between the center of the visual axis and the lens center was 0.33 ± 0.20 mm, which was like the corresponding angle alpha data (0.33 ± 0.14 mm) obtained by Fu using iTrace, the two values are not the same [13].

Although these authors found no significant correlation between the angle alpha and certain objective parameters of visual quality after the implantation of multifocal IOLs, other researchers continue to think that the angle alpha is an important factor influencing the selection of premium IOLs. Moreover, the Dong group emphasized that the angle alpha data calculated from the distance between the center of the limbus (recognized as the central point of the lens) and the visual axis in some clinical devices should be updated in the future [11].

Li et al. investigated age-related lens tilt and decentration by SS-OCT (CASIA2^®^, Tomey Corp., Nagoya, Japan) in 230 participants aged between 7 and 90 years [14]. The main study findings indicated that the lens had a mean tilt toward the inferotemporal direction of 4.3 ± 1.5° (range: 0.7 to 8.95°). The mean decentration towards the superotemporal direction was 0.17 ± 0.12 mm (range 0.03 to 1.15 mm). Thus, it was observed that lens tilt was greater in eyes with wider α angle and narrower anterior chamber depth (ACD), whereas lens decentration was greater in younger eyes with wider κ and α angles.

Two studies have assessed lens subluxation using OCT [15,16]. It is known that this condition can be challenging to diagnose if clinical signs are subtle, such as the absence of phacodonesis or a very slight tilt or decentration of the lens. In this context, the use of OCT can be beneficial for measuring ACD, the relative position of the lens, and lens tilt. These parameters are crucial for differentiating subluxation from other conditions that may cause primary angle closure, such as a low relative lens position (RLP), which could indicate anterior subluxation that, if not detected and managed appropriately, might lead to severe complications such as angle-closure glaucoma due to pupillary block. OCT could assist not only in diagnosing this condition but also in planning surgical interventions more safely, as it allows for the evaluation of zonular integrity and the precise localization of the displaced lens.

Tang et al. evaluated new diagnostic indicators for acute angle closure secondary to lens subluxation using CASIA 2 OCT [15]. The results showed that parameters such as lens vault (LV), the anterior radius of pronounced curvature of the lens, and ACD had a high predictive power (AUC = 0.87, 0.89, and 0.86, respectively). The combination of LV, tilt, and tilt axis in a mathematical model demonstrated very high diagnostic power (AUC = 0.98), suggesting that these indicators may improve the detection and treatment of acute angle closure secondary to lens subluxation, providing a better understanding of the pathogenetic role of zonulopathy in angle-closure glaucoma.

In addition, Xing et al. analyzed anterior lens subluxation, which is often mistaken for primary angle-closure glaucoma [16]. The article described how OCT can be used to diagnose these hidden subluxations. The measurement of ACD and RLP are key elements for diagnosis. The authors emphasize that an ACD of less than 1.8 mm or an asymmetry greater than 0.2 mm between the two eyes may be indicative of lens subluxation. Additionally, the RLP value demonstrated a high sensitivity (AUROC: 0.934) for distinguishing cases of acute angle closure secondary to lens subluxation versus other causes.

### 3.4. Age-Related and Accommodation-Related Changes

Different OCT-based studies have analyzed age-related and accommodation-related changes in the lens.

Shaoy et al. investigated how the biometry of the anterior segment of the eye changes with age and during accommodation, as detected by OCT. They observed that with aging, the lens becomes thicker (i.e., increases on average 0.024 mm per decade), while the ACD decreases (on average 0.1 mm per decade) [17]. Similarly, Richdale et al. analyzed the effect of age, accommodation, and refractive error in the adult eye using OCT. They demonstrated that accommodative capacity decreases significantly with age (averaging 0.4 diopters per year after age 40), due to the increased stiffness and thickness of the lens [18]. Waring et al. correlated intraoperative measurements of lens diameter, thickness, and volume with biometry and age using OCT [19]. They found that lens diameter increases by approximately 0.02 mm per decade, while thickness increases by 0.03 mm per decade, which may impact accommodation. Neri et al., using SS-OCT, demonstrated that OCT can capture real-time changes in lens thickness and curvature during accommodation [20]. They highlighted that in young subjects, the lens can change its thickness by approximately 0.3 mm during accommodation, while in older subjects this change is much less pronounced, reflecting the decreased accommodative capacity due to presbyopia. These data therefore underscored the utility of OCT for detecting and quantifying age- and accommodation-related changes in the lens.

### 3.5. OCT in Cataracts

#### 3.5.1. Cataract Grade Classification

To date, the gold standard classification for cataracts is the Lens Opacity Classification System III (LOCS III) [2]. In this system, the different characteristics of the cataract are classified during slit-lamp examination according to standard photographs that serve as a guide. Although it is widely used in research contexts, its clinical application is limited by the subjective nature of the assessment and by the fact that it is a laborious procedure, which hampers its routine implementation.

With the advent of new anterior segment imaging devices, such as aberrometry, double-pass systems, and tomography, it has become possible to perform objective measurements of the internal densities of the lens.

Numerous studies have found significant correlations between nuclear opacity measured with LOCS III and/or corrected distance visual acuity (BCVA) and different parameters from both double-pass systems, such as the Dysfunctional Lens Index (DLI) [21] or the Objective Scatter Index (OSI) [22,23], as well as lens densitometry measured with Scheimpflug tomography [21,24,25,26,27,28]. This opens the possibility for categorizing (classifying) the degree of lens involvement. Classifications based on parameters that measure scattering such as the OSI have been described [29], although their implementation in daily practice is limited primarily due to their dependence on the level of technology penetration in ophthalmological centers.

On the other hand, one of the most studied classifications has been proposed with the Pentacam software (the Pentacam Nucleus Staging or PNS Version 1.30r04, OCULUS, Inc, Wetzlar, Germany), which has five degrees of severity of nuclear opacity and correlates better than other classifications with intraoperative parameters, particularly in nuclear cataracts [30]. However, due to its moderate image resolution and limited penetration, the posterior layers may not be visible, and measurements may be affected by artifacts in the presence of cortical opacities [1,31].

Due to its longer wavelength, which results in a greater penetration in deeper tissues, even in the presence of opacities, and its higher image resolution, SS-OCT has addressed the limitations of Scheimpflug tomography [31]. Additionally, it is a technique that is less affected by external illumination, is faster, and does not require pupil dilation for the assessment of the posterior layers of the lens.

Thus, a good correlation has also been found between lens density measured with SS-OCT and the LOCS III system (particularly for the nuclear component) [2,32,33,34,35], with BCVA [31,36], and with surgical parameters [31,33,36].

Mackenbrock et al. quantitatively assessed lens opacity using an SS-OCT ANTERION (Heidelberg Engineering GmbH, Heidelberg, Germany) device to establish its correlation with images obtained via Scheimpflug camera Pentacam AXL Wave (OCULUS Optikgeräte GmbH, Wetzlar, Germany) [31]. In this prospective and cross-sectional study, 51 patients with cataracts were included. Lens densitometry was analyzed automatically using customized software (MATLAB (Version R2021b, MathWorks, Natick, MA, USA) to assess overall lens density, nuclear density, and linear density. Statistically significant correlations were found between SS-OCT and the Scheimpflug camera in the analysis of global lens density (ρ = 0.47, *p* < 0.001) and in the analysis of nuclear lens density (ρ = 0.73, *p* < 0.001). The study demonstrated good agreement between the devices in lens densitometry, with SS-OCT providing superior imaging of the lens compared to the Scheimpflug device and showing greater correlation with different clinical parameters. The authors concluded that these findings suggest that high-resolution SS-OCT might be a preferable option for automatic cataract grading and preoperative planning [31].

Based on these characteristics, in 2017, Panthier et al. published a study that included 285 eyes from 155 patients. This study found that mean lens densitometry (ALD) measurements were repeatable and significantly correlated with other assessment methods, such as the ocular scatter index (OSI) and the Pentacam Nucleus Staging (PNS) score [37]. An ALD greater than 73.8 pixel units was identified as the threshold for cataracts, with a sensitivity of 96.2% and specificity of 91.3%. The combination of ALD with visual acuity showed an area under the ROC curve of 0.975, highlighting its potential as a reliable tool for determining the necessity of surgery in symptomatic patients.

One year later, in a similar study, Bras et al. developed an SS-OCT-based cataract nucleus hardness grading system and compared it with the conventional subjective LOCS II grading [38]. The study included 113 patients (186 eyes, 40 controls without cataracts and 146 with cataracts). The cataracts were graded according to the nucleus score as mild (41 to 65), moderate (66 to 90), and dense (greater than 90). A good correlation of 0.86 and 0.76 was observed for two independent examiners between the values obtained by the SS-OCT system and the LOCS II classification. Furthermore, the SS-OCT-based system showed a significant correlation with the time and energy required for phacoemulsification, suggesting that this method could be useful for planning cataract surgery [38].

Based on the classification of Bras et al., [38] Wu et al. [36] made a minor modification to the scale and compared it with LOCS III, as well as with intraoperative parameters, demonstrating the validity of the classification due to the correlations with both parameters.

De Castro et al. performed a study using a new SS-OCT laboratory prototype designed to enhance the visualization of the lens. According to the results of the study, this SS-OCT was able to reveal the presence of cortical or subcapsular cataracts, observed as linear areas with increased reflectivity [39]. In addition, they observed that the OCT signal in the lens nucleus correlated with the clinical grade of the nuclear cataract.

One of the primary objectives of OCT application in the assessment of the lens is to establish an objective and standardized system for classifying the degree or severity of cataracts [2]. Ideally, an effective system should correlate with functional metrics, demonstrate high reproducibility, and facilitate clinical decision-making.

Furthermore, it should be user-friendly and fast, so as not to interfere with clinical routines or cause inconvenience to the patient. Finally, the standardization of a classification system would improve scientific research and communication among researchers, as well as epidemiological studies [2]. All these aspects have already been demonstrated by OCT, with different software options currently available that can offer objective measurements of the opacity or reflectivity of the lens (Figure 2). However, the correlation with functional parameters remains the primary focus of current research. Thus, OCT is positioned as a tool that could enable the monitoring of cataract progression by analyzing changes in the lens regarding both the size and the distribution of opacities, as well as in selecting the optimal timing for surgery, thereby improving the clinical management of complex cases.

Kling et al. highlighted the need for a method to assess the biological age of a patient’s lens, as this could enable a more objective decision-making process regarding the most appropriate type of refractive surgery for pre-presbyopic patients aged 45 to 55 years [40]. As a key element of ocular accommodation, the inherent mechanical stiffness gradient and the gradient refractive index (GRIN) of the lens determine its deformability and optical functionality. Consequently, the authors proposed the use of OCT elastography to quantify the GRIN profile and the deformation characteristics of the lens, as it has the potential to enhance the diagnosis and monitoring of lens disorders, as well as to guide future refractive interventions.

For all these reasons, cataract classification systems based on technologies such as OCT outperform subjective systems based on slit-lamp examination, which can be prone to errors and time-consuming. However, their implementation remains a significant challenge and is primarily utilized in research settings.

#### 3.5.2. Correlation of Cataract Severity Degree with Surgical Parameters

Numerous studies have demonstrated that the Pentacam PNS correlates better with accumulated dissipated energy (US) than LOCS III [25,26], as well as with balanced salt solution (BSS) consumption and ultrasound time (U/S) [26,30].

Using different automatic lens opacity detection software, including OCT-based studies, has revealed a strong correlation with functional and surgical parameters such as visual acuity, phacoemulsification energy, and the duration of the surgical procedure [31].

Overall, the strongest correlation with functional metrics was observed when analyzing the lens nucleus, utilizing both Scheimpflug-based devices and OCT. Heyworth et al. concluded that the rigidity of the lens primarily originates from the nucleus; thus, it is logical that cortical cataracts have minimal impact on phacoemulsification energy [41]. Similarly, Mackenbrock et al. found a significant correlation between SS-OCT and cumulative dissipated energy (CDE) during cataract surgery (r = 0.57, *p* < 0.001) [31]. However, it is important to note that while the degree of opacity assessed through imaging techniques serves as an indicator of lens hardness, phacoemulsification power remains influenced by diverse factors, including the surgeon’s experience and preferences. Moreover, although objective measurements of preoperative opacity and phacoemulsification energy can be easily calculated, establishing a link to visual acuity and the patient’s subjective perception is considerably more complex.

#### 3.5.3. The Role of Artificial Intelligence in Cataract Classification

Recently, the development of deep learning networks has enhanced the imaging capabilities of imaging devices for the detection and classification of cataracts, improving interpretative accuracy and increasing the robustness of these classification systems when applied to cases that deviate from the norm. These classification systems, which can be used for both screening and preoperative diagnoses, are valuable for prospective studies but still require implementation and validation in daily clinical practice [2].

Zhang et al. developed a deep learning algorithm that utilized fundus images to classify cataracts, achieving an accuracy of 94.75% [42]. As lens opacity increases, the sharpness of the fundus image decreases, paralleling the loss of visual acuity experienced by the patient. Although this provided a good indication of cataract severity, it is also influenced by other ocular opacities (such as those of the cornea and vitreous) as well as camera settings. Furthermore, this method has the significant limitation of being unable to differentiate between nuclear, cortical, or posterior subcapsular opacities.

A more common approach is to use still slit-lamp images and retro-illuminated images to train a deep learning network based on the LOCS III scale. Using the Deep Lens Net framework, Keenan et al. showed that, for the two most prevalent types of cataracts—nuclear and cortical—the deep learning method exhibited strong performance [43].

However, this has been surpassed by studies such as that of Zéboulon et al., who used OCT scans to classify lens opacities, and in which they implemented a deep learning tool for use in clinical practice, achieving a sensitivity of 94.4% and a specificity of 94.7% [44]. One of the main criticisms of deep learning methods is that they are often considered as a “black box” because they do not elucidate how the predictions are obtained, making the interpretation of results challenging. It is noteworthy that in the work of Zéboulon et al., probability maps are provided overlaid on the OCT scans that are easy to understand and show the areas with the different degrees of cataract opacification [44].

Other studies have employed a subtype of convolutional neural network known as the “Adaptive Feature Squeeze Network” to improve the performance of cataract classification using AS-OCT. While they achieve improvements, the classification has only three levels and is only applicable to the nucleus [45]. However, there are already articles with promising results in the assessment of the cortical region [46].

It is important to note that these systems depend on a large volume of data and accurate labeling of images, as they depend on predefined landmarks to recognize the structures of the lens, making them susceptible to errors when examining patients with characteristics that deviate from the norm (such as patients with uveitis and synechiae).

#### 3.5.4. Intraoperative OCT for Lens Evaluation

The emergence of intraoperative OCT (iOCT) has contributed to more precise, safe, and complex ophthalmic surgical maneuvers, and it has recently been used in the field of cataract surgery.

Odden et al. described the utility of iOCT in pediatric cataracts under specific circumstances, including maneuvers such as the removal of posterior capsule opacification that could not be performed using YAG laser capsulotomy; the release of posttraumatic anterior and posterior synechiae in opaque corneas; the surgical removal of epithelial cell proliferation in the lens following pediatric cataract extraction with IOL implantation; and the identification of the integrity or defects of the posterior capsule [47].

On the other hand, Tassignon and Van Os have analyzed, using iOCT, the relationship between the anterior hyaloid and the posterior capsule of the lens, describing the normal anatomy of this interface in children, and demonstrating its variation in different forms of pediatric cataracts, such as posterior plaques and posterior capsule opacification due to abnormal adhesion to the anterior hyaloid [48]. The authors conclude that understanding these differences will assist surgeons in managing pediatric cataracts more safely and with greater confidence.

Another interesting study was conducted by Chen et al., who investigated the morphology of the posterior cortex of the lens and the posterior capsule (PC) in pediatric patients with posterior lens opacities using iOCT [49]. A total of 62 eyes from 53 patients, with a mean age of 3.8 years, were included. Four morphological variants of posterior lens opacity were observed: Type I (54.8%): intact PC; Type II (32.3%): intact PC that protrudes into the anterior vitreous; Type III (4.8%): deficient PC with an inability to delineate the PC; and Type IV (8.1%): dense opacity with an inability to characterize the posterior cortex and the PC. In terms of practical application, phacoemulsification was feasible in Types I and II. For Types III and IV, manual nucleus extraction was performed instead of phacoemulsification. The dehiscence of the PC was observed in three cases (100%) of Type III during surgery, while no cases of dehiscence were noted in the other types. The authors concluded that the morphology of the PC and the posterior cortex of the lens in pediatric cataracts can be categorized, and the integrity of the PC can be assessed using iOCT, which is useful for guiding surgical strategies and preventing complications in cases with pre-existing defects of the PC.

Regarding posterior polar cataract, Pujari et al. studied a total of 12 eyes from 12 patients with clinically confirmed posterior polar cataract who underwent cataract surgery performed by a single surgeon under the direct guidance of iOCT [50]. The study focused on the changes at the nuclear–epinuclear junction and the opacity–capsule junction during and after hydrodelineation, as well as the changes in the capsular junction after nucleus extraction and their dynamic alterations throughout the surgical procedure. In terms of results, with regular hydrodelineation, optimal separation of the nuclear–epinuclear layer was observed in 11 patients. Once a golden ring was achieved through the hydrodelineation procedure, repeated attempts could be made within it to reduce the likelihood of capsular damage. During this maneuver, several observations were observed, including the fracture of the posterior opacity with tension on the underlying capsule, inadvertent hydrodissection during hydrodelineation, continuous distension of the posterior capsule, and ruptures of the posterior capsule, even with precise and standardized surgical maneuvers. The authors concluded that iOCT provided a better understanding of real-time changes in the lens structure during posterior polar cataract surgery, which may help minimize inadvertent complications.

Another situation where iOCT has represented a significant advancement is in the behavior of white cataracts, where the presence of elevated intracapsular pressure can precipitate complications during capsulorhexis (such as the “Argentine flag” sign). In this context, Titiyal et al. investigated the behavior and morphology of 50 white cataracts using iOCT [51]. They described five types of white cataracts defined according to two observed morphological characteristics: (a) the convexity and behavior of the anterior capsule and (b) the morphology of the anterior cortex.

The classification proposes a relative risk of peripheral extension of the capsulorhexis based on the morphology observed in iOCT, allowing the surgeon to implement corrective maneuvers even before performing the initial paracentesis and decompressing the globe.

### 3.6. Clinical Utility of OCT in Different Lens-Related Pathologies

**Figure 3:**
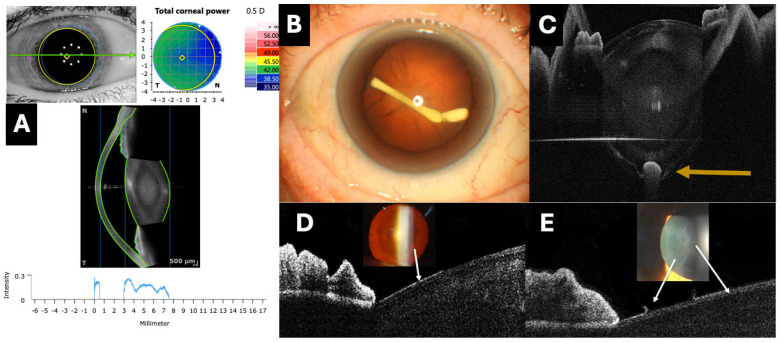
Clinical applications of optical coherence tomography (OCT) in lens evaluation. (**A**) A software program that quantifies the reflectivity of the crystalline lens using a graph (Anterion AS-OCT, Heidelberg engineering, Heidelberg, Germany). (**B**,**C**) The intracrystalline injection of an intravitreal dexamethasone implant inside the posterior capsule (orange arrow) imaged with the PlexElite 9000 OCT with an AS adapter lens (Carl Zeiss, Jena, Germany). (**D**,**E**) Hyperreflective plaques (white arrows) on the anterior surface of the lens assessed by the RTVue OCT with an AS adapter lens (Optovue, Fremont, CA, USA), corresponding to pseudoexfoliative material.

The utility of OCT in the analysis of the lens has been described in diverse clinical scenarios (Figure 1, Figure 2 and Figure 3).

#### 3.6.1. Traumatic Cataracts

Another potential application of OCT is to assess the status of the PC in complex cases such as traumatic cataracts, as it is crucial to avoid unintentional tears of the posterior capsule and the loss of lens fragments into the vitreous cavity during surgery [52]. Compared to the zonule, the PC can be particularly thin, complicating its evaluation with UBM. Therefore, Tabatabaei et al. evaluated the accuracy of UBM and OCT in detecting defects in the posterior capsule in cases of traumatic cataracts [53]. They reported that the positive and negative predictive values for UBM were 70.4% and 73.3%, respectively, while for OCT they were 75.5% and 95.5%. Thus, although both methods are effective, OCT appears to be superior to UBM for this application [52,53].

#### 3.6.2. Lifebuoy Ring Cataract

Koshiishi et al. described the use of OCT for the analysis of lifebuoy ring cataract [54]. Due to its low frequency, there are limited reports on treatment, and no standardized surgical approaches exist. Slit-lamp examination revealed central calcification of the lens capsule and slightly opaque cortical tissue at the periphery, with no observable lens nucleus. OCT (CASIA2, TOMEY^®^, Nagoya, Japan) displayed a typical image showing fusion of the anterior and posterior capsules and the absence of the lens nucleus.

#### 3.6.3. Pseudoexfoliation Syndrome

Our group described the usefulness of OCT for evaluating the presence of residual pseudoexfoliation (PSX) material on the anterior surface of the lens, observed as irregular hyperreflective lines (Figure 2) [55]. The primary utility would be to provide a diagnostic confirmation in cases with early or uncertain signs.

#### 3.6.4. Lens Abscess

Kaur and Gurnani have described the utility of OCT for the evaluation of a lens abscess [56]. Examination with a slit lamp typically reveals a heterogeneous opacity of the lens or a lenticular cavity filled with pus. Techniques that allow for the effective evaluation of an abscess in the lens include B-mode ultrasound, OCT, and UBM. It is crucial to differentiate lens abscesses from traumatic cataracts, as the implantation of an intraocular lens during the first procedure is contraindicated.

#### 3.6.5. Assessment of the Posterior Capsule of the Lens Concerning Intravitreal Injections

Given the increasing frequency of intravitreal injections, there are instances where posterior capsule ruptures can be detected due to improper injection techniques, including contact with the injection needle. There have even been cases of intracrystalline placement of the sustained-release dexamethasone implant, such as the case shown in Figure 2 [57].

#### 3.6.6. Anterior and Posterior Lenticonus

Lenticonus, both anterior and posterior, is a congenital anomaly of the lens characterized by a conical protrusion of its surface, which can lead to significant visual distortion [58]. OCT enables the detailed visualization of lens morphology and an accurate quantification of the conical protrusion, allowing for the detection of lens thinning around the protrusion and alterations in the curvature of the anterior or posterior capsule.

### 3.7. Clinical Usefulness of OCT in Study of Pseudophakic IOLs

**Figure 4:**
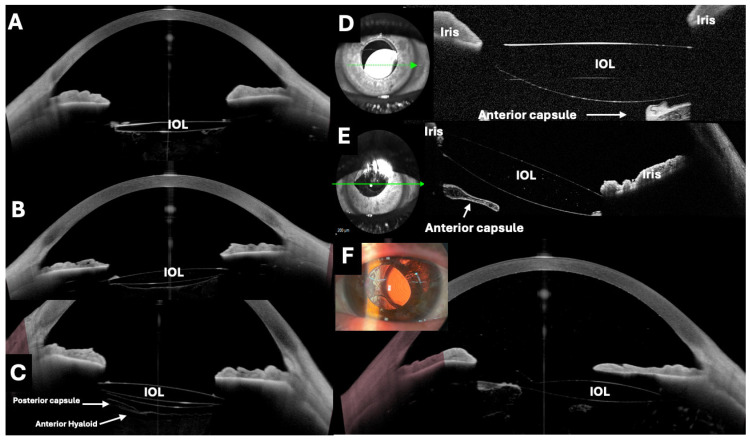
Different examples of intraocular lenses (IOLs) in the capsular bag evaluated by optical coherence tomography (OCT). (**A**–**C**,**F**) Images taken with the Anterion AS-OCT (Heidelberg engineering, Heidelberg, Germany), with D and E taken with the RTVue OCT with an AS adapter lens (Optovue, Inc., Fremont, CA, USA). It is possible to assess the centering, inclination, and state of the lens, as well as the neighborhood relationships. (**A**) The IOL and a capsulotomy. (**B**) The entire posterior capsule. (**C**) The anterior and posterior surfaces of the IOL, the posterior capsule, and the anterior hyaloid can be seen simultaneously. (**D**) Subluxated IOL towards the inferonasal quadrant. (**E**) The IOL optic is partially above the iris rim, which produces a tilt of the lens and a pupillary corectopia. (**F**) The OCT demonstrates a significant decentration. The OCT facilitated the monitoring of the patient follow-up.

**Figure 5:**
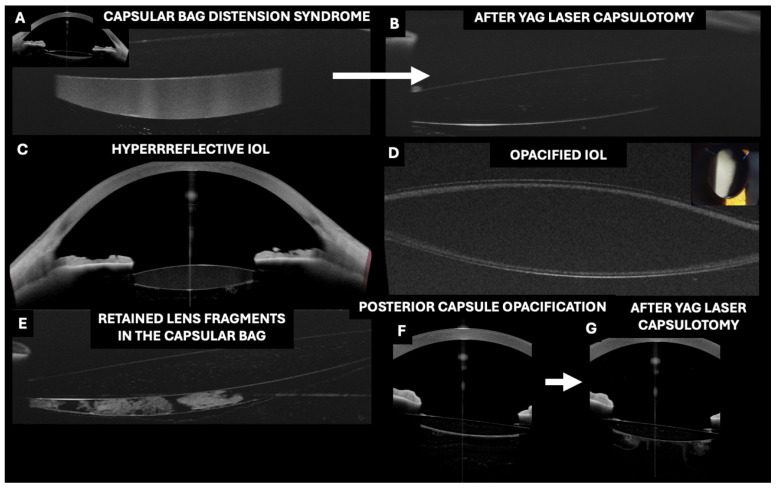
Applications of optical coherence tomography (OCT) in the evaluation of pseudophakic intraocular lenses (IOLs) inserted in the capsular bag. (**A**–**C**,**F**,**G**) Images taken with the Anterion AS-OCT (Heidelberg engineering, Heidelberg, Germany) and (**D**,**E**) with the RTVue OCT with an AS adapter lens (Optovue).

**Figure 6:**
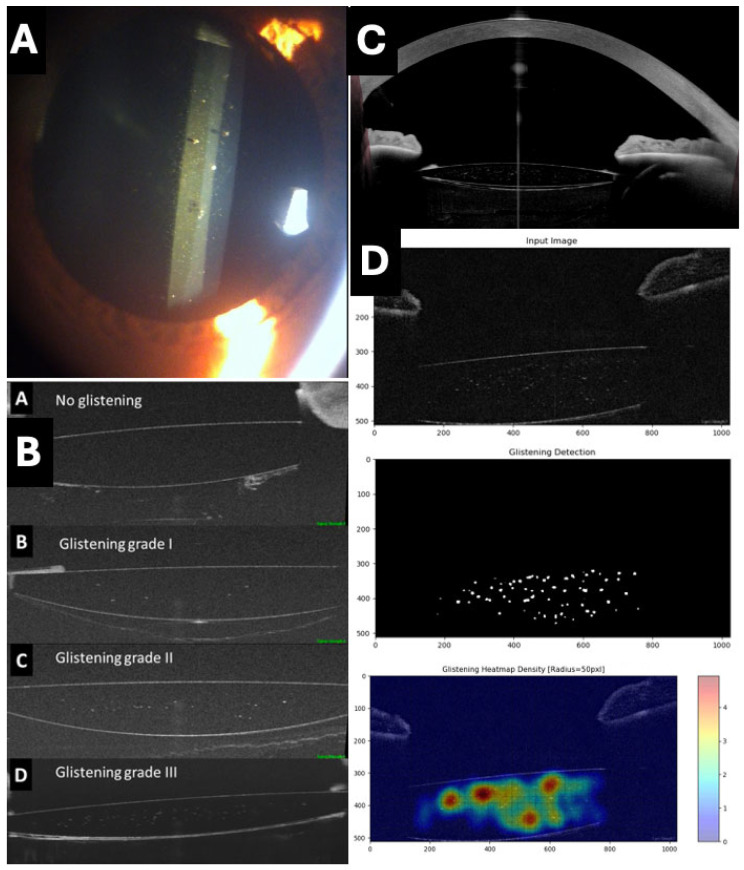
Assessment of glistening in intraocular lenses (IOLs). (**A**) Typical appearance of glistening on slit lamp. (**B**) Proposed classification of glistening (from A: no glistening to D: grade III) using RTVue OCT with AS adapter lens (Optovue) (**C**) IOL with glistening and its relationship with rest of structures of anterior chamber obtained with Anterion AS-OCT (Heidelberg engineering, Heidelberg, Germany). (**D**) Assessment and quantification of glistening using a deep learning artificial intelligence system.

In recent years, the usefulness of OCT in the assessment of pseudophakic IOLs in different clinical scenarios has been described (Table 1, Figure 4, Figure 5 and Figure 6).

### 3.8. OCT in the Evaluation of IOL Glistening

The phenomenon of IOLs glistening is characterized by the formation of tiny microvacuoles (MVs) filled with water within the IOL material, leading to light scattering and generating a sparkling or glowing effect [59]. This phenomenon is common across various materials and models of IOLs and has been the subject of investigation in recent years to understand its incidence, risk factors, progression, and potential clinical impact.

Traditionally, glistening has been investigated in vitro in laboratories or by slit-lamp photography. Recently, our group has used OCT as a simpler alternative for the analysis of IOL glistening, demonstrating it as a straightforward, reproducible, and objective method by identifying hyperreflective points within the IOL optics. This approach allows for the satisfactory evaluation of different types and models of IOLs (Figure 6) [60,61]. In addition, our group described the use of a deep learning algorithm for the automatic detection and quantification of glistening (Figure 6) [61].

In contrast to the MVs associated with glistening, pigment deposits or pseudoexfoliative materials are easily identifiable as they are located on the IOL surface. Additionally, PC opacification or remnants of masses are clearly visualized between the posterior surface of the IOL and the PC of the bag (Figure 5).

### 3.9. OCT in the Evaluation of IOL Opacity

OCT has been shown to be useful for detecting IOL opacification (Figure 5). Yildirim et al. proposed a novel methodology to assess IOL opacification using OCT (Anterion, Heidelberg Engineering, Heidelberg, Germany) [62]. The authors concluded that this high-resolution OCT imaging technique can be used to visualize IOL opacities. The degree of opacification was well correlated with lens-induced glare. OCT could potentially be used in the future as a tool to predict visual impairment and assist clinicians in quantifying and understanding patient complaints, particularly in cases where coexisting ocular pathologies complicate functional vision testing.

### 3.10. OCT in the Evaluation of Capsular Distension Syndrome

Postoperative capsular distention syndrome (CBDS) is a rare complication of cataract surgery that can occur both in the early postoperative period and several years after an uncomplicated surgery [63,64]. Kanclerz et al. reported that the visualization of CBDS can be facilitated by Scheimpflug and BMU imaging. However, these methods lack a high resolution, and due to the short wavelength of Scheimpflug imaging (475 nm), visualizing the posterior capsule may be impossible [63,64]. In contrast, OCT allows the visualization of the highly reflective fluid between the IOL and the PC, accurately quantifying the degree of bag distention (Figure 5). Using this technique, it is possible to visualize the change after Nd:YAG laser posterior capsulotomy.

### 3.11. OCT in the Evaluation of Toric IOLs

Lucisano et al. described the use of a simple tool for evaluating postoperative alignment of toric IOLs based on OCT [65], demonstrating that the axis alignment of a toric IOL can be assessed simultaneously with the topographic astigmatic axis. This method eliminates potential errors resulting from head tilt and shows a strong correlation with the astigmatic correction achieved.

### 3.12. OCT in the Evaluation of IOL Decentration and Tilting

Although advances in surgical techniques and IOL design have significantly improved the issue of proper IOL centration, accurate alignment (tilt and decentration) of the pseudophakic IOL remains essential for optimizing optical performance in terms of astigmatism, BCVA, and higher-order aberrations. Several studies have used OCT to visualize the position of the IOL and assess postoperative IOL tilt (Figure 4) relative to the limbus, but there are very few studies on estimating postoperative IOL decentration with OCT using a 3D reconstruction method. In this context, Wang et al. performed a study to evaluate IOL tilt and decentration using OCT [10]. The authors concluded that OCT can be used as an alternative for the analysis of IOL tilt and decentration through 3D reconstruction.

It is important to note that accurately assessing IOL malposition is crucial in the postoperative follow-up of cataract surgery, as it can lead to refractive errors and retinal image problems, impacting negatively on visual acuity. In some cases, these issues may indicate the need for IOL exchange, repositioning, or removal. Measurements of tilt angle and decentration metrics have been shown to be both repeatable and reproducible, indicating that OCT can be used as an alternative for analyzing IOL tilt and decentration using the 3D reconstruction method.

Wasery et al. published a prospective study that aimed to predict postoperative IOL tilt using preoperative biometric data and a machine learning-based approach, with measurements performed via OCT [66]. The study concluded that postoperative IOL tilt can be predicted with high accuracy using preoperative biometric data. The most relevant parameters for prediction were preoperative IOL tilt, pupil decentration, lens thickness, axial length of the eye, and IOL decentration. This model, based on a combination of partial least squares regression and machine learning, demonstrated excellent potential for improving postoperative refractive outcomes by accurately anticipating IOL tilt.

### 3.13. Effective Lens Position Using OCT

OCT has emerged as an effective tool for analyzing effective lens position (ELP) (Figure 3).

Langenbucher et al. evaluated the ability of OCT to accurately predict postoperative IOL decentration and tilt [67].

The authors concluded that AS-OCT is an extremely accurate tool for predicting the final position of the IOL, allowing surgeons to anticipate and correct IOL positioning issues, thereby reducing the risk of postoperative complications.

Gouvea et al. evaluated the accuracy of spectral-domain OCT in predicting the preoperative and postoperative meridional position of IOLs, defined as the distance from the corneal epithelium to the lens equator. They compared the postoperative anatomical position of the lens (defined as the distance from the corneal epithelium to the surface of the IOL) with the preoperative meridional position to calculate their correlation and mean difference [68]. The authors concluded that spectral-domain OCT provides a high accuracy in predicting the meridional position of the IOL, improving surgical planning and postoperative visual outcomes by reducing the margin of error in IOL placement.

João et al. analyzed changes in IOL position and anterior chamber parameters following Nd:YAG laser posterior capsulotomy using OCT [69]. They observed a mean ACD reduction of 0.1 mm and an IOL tilt of up to 2.5 degrees, highlighting OCT’s ability to detect and address subtle postoperative changes (Figure 4).

Wu et al. investigated the ability of OCT to predict the ELP in patients with angle closure, comparing the effective ELP with predictions based on measurements obtained with OCT [70]. The results showed that AS-OCT achieved a precision of ±0.2 mm in predicting the IOL position, improving the accuracy of IOL calculation formulas in eyes with complex anatomical features. The authors concluded that AS-OCT provides superior accuracy in predicting the IOL position in cases of angle closure, allowing for better surgical planning and more accurate refractive outcomes in patients with a complex ocular anatomy. Additionally, the authors also observed that axial length and lens thickness were key determinants of the final IOL position. The results of this study suggested that adjusting axial length and lens thickness in preoperative calculations can significantly improve the final IOL position and refractive outcomes, highlighting the importance of considering these parameters in surgical planning [70].

Yoo et al. proposed the introduction and evaluation of a new formula based on 3D OCT to improve the prediction of IOL position [71]. They developed an innovative formula that used 3D OCT data to calculate IOL position and compared the mean prediction error between the new formula and traditional formulas. This resulted in a reduction in the mean prediction error from 0.5 D to 0.25 D with the new formula compared to traditional methods, thereby improving surgical planning and postoperative visual outcomes. Therefore, the 3D OCT-based formula significantly improved the accuracy of IOL position prediction, optimizing surgical planning and potentially leading to better visual outcomes for patients.

Similarly, Ding et al. quantified the tilt and axial stability of the postoperative IOL using high-speed SS-OCT [72]. IOL stability was quantified with a standard deviation of 0.05 mm in axial positioning and 0.5 degrees in postoperative tilt, demonstrating the capability of OCT to provide an accurate three-dimensional assessment of IOL stability. The study concluded that high-speed SS-OCT offered an extremely accurate evaluation of postoperative IOL stability, allowing surgeons to monitor and adjust lens positioning with high precision to ensure optimal outcomes.

In a study that applied machine learning models to predict IOL tilt with high accuracy, Waser et al. found that the model predicted IOL tilt with an accuracy of 93%, which appeared to significantly improve preoperative planning [66]. The authors concluded that the integration of machine learning models with OCT could provide remarkable accuracy in predicting IOL tilt, potentially revolutionizing surgical planning and minimizing errors in lens placement.

These studies demonstrated how the use of OCT and advanced analysis techniques contribute to greater precision in cataract surgery, optimizing IOL placement and reducing postoperative complications.

### 3.14. Negative Dysphotopsia

Another important topic that needs to be discussed is negative dysphotopsia [73].

This may be defined as a visual phenomenon in which patients perceive a shadow or dark area in the temporal region of the visual field following uneventful cataract surgery with IOL implantation in the capsular bag. Different theories attempt to explain the origin of this complication. Vamosi et al. described by UBM that a possible cause could be an increased distance between the iris and the IOL, suggesting that reducing this distance might lead to a remission of the symptoms [74]. The main therapeutic options include implanting an additional IOL in the sulcus (piggyback) alongside the previous IOL, or reverse optic capture, in which the haptics remain in the capsular bag while repositioning the IOL optic anteriorly into the sulcus, capturing its edge with the capsular bag. These maneuvers may reduce negative dysphotopsia. In this regard, OCT allows for the analysis of the relationship between the IOL and surrounding structures (Figure 3), which could be useful in this complex clinical scenario and in evaluating changes following the new surgery.

### 3.15. Limitations of OCT in the Evaluation of the Crystalline Lens and Pseudophakic IOLs

While SS-OCT provides the benefit of high-resolution volumetric data, the study’s limitations stem from corneal specular reflections, which cause image saturation, and the inability to visualize lens regions posterior to the iris. Consequently, the zonules and peripheral lens areas, where initial opacities may develop, remain inaccessible for imaging [38].

Another limitation of OCT is its image resolution, typically ranging from 5 to 8 microns axially and 20 microns transversely, which may lead to an underestimation of the number of microvacuoles in the glistening, particularly if their size falls below this threshold. Additionally, image quality is highly dependent on factors such as artifacts caused by ocular media opacities or eye movements during acquisition. Furthermore, the processing of background noise by the device’s software can also impact overall image clarity.

## 4. Conclusions

Over the last years, OCT has become a valuable tool in clinical practice for assessing the lens and pseudophakic IOLs, allowing a detailed and non-invasive evaluation of these structures, and their spatial relationships. Its clinical utility has been demonstrated in analyzing the lens’s shape, position, and size, as well as in assessing the type and severity of cataracts and their different components. While qualitative analysis of the lens is highly beneficial, the ability to quantitatively measure and grade its features is currently reproducible only for the nucleus. The main actual applications of lens evaluation with OCT focus on improving the detection of visual disturbances related to incipient crystalline changes, refining biometry refractive results in uncommon cases, and preoperative planning based on crystalline lens morphology and position to prevent surgery-related complications.

Regarding pseudophakic IOLs, it allows accurate assessment of lens positioning and centration (effective lens position, toric IOL alignment and tilt), as well as the identification of potential complications such as glistening, IOL or posterior capsule opacification, and late capsular distension syndrome. The main actual applications focus on improving the detection of subtle IOL changes that may justify visual disturbances, sometimes impossible to detect with other imaging methods, aiding in applying appropriate treatment. For these reasons, we think that OCT provides critical diagnostic insights and should be progressively integrated into the diagnostic framework for cataracts and intraocular lenses.

## Figures and Tables

**Table 1:** Main uses of optical coherence tomography (OCT) for assessing lens and pseudophakic intraocular lenses (IOLs).

**Crystalline Lens Evaluation by OCT**
Morphology, positioning, and size of the lens
Changes with age and accommodation
Assessment of the degree of cataract: degree of reflectivity and density of the different components of the lens (cortical, nuclear, subcapsular, polar, etc.)
Assessment of complex cataracts: white or intumescent cataract, brunescent or hypermature cataract, traumatic cataract
Assessment of the cataract hardness (since it correlates with surgical time and energy)
Detecting the presence of pseudoexfoliative material
Assessment in other clinical situations: integrity of the posterior capsule after intravitreal injections, anterior and posterior lenticonus, lens abscess, etc.
**Pseudophakic IOL Evaluation by OCT**
Shape, positioning (centering), and tilting of IOLs
Relationship of the IOL to the anterior and posterior capsule, anterior hyaloid, and iris
Presence of posterior capsular opacity, mass remnants, capsular block syndrome, or late capsular distension syndrome
Presence of glistening or IOL opacities
ELP assessment

OCT: optical coherence tomography; IOL: intraocular lens; ELP: effective lens position.

## Data Availability

Data sharing is not applicable.
